# Mechanisms and therapeutic strategies of copper homeostasis in the pathogenesis of sepsis-induced cardiomyopathy

**DOI:** 10.3389/fcell.2026.1753386

**Published:** 2026-03-05

**Authors:** Zihao Xie, He Wang, Bohua You, Mengmeng Li, Duo Li, Huaying Wu

**Affiliations:** 1 Hunan Normal University Health Science Center, Changsha, Hunan, China; 2 Key Laboratory of Study and Discovery of Small Targeted Molecules of Hunan Province, Hunan Normal University Health Science Center, Changsha, Hunan, China; 3 Department of General Surgery, People’s Hospital of Honghu City, Honghu, Hubei, China

**Keywords:** cell death, copper homeostasis, cuproptosis, sepsis, sepsis-induced cardiomyopathy

## Abstract

Sepsis-induced cardiomyopathy (SCM) is a severe, mortality-increasing sepsis complication, with copper homeostasis imbalance as a key pathogenic factor. Copper (Cu) plays a dual role: as an essential enzyme cofactor, it regulates vital processes including energy metabolism and redox balance; however, both excess and deficiency disrupt cellular homeostasis and induce cardiomyocyte injury. This review summarizes core pathophysiological mechanisms linking copper homeostasis imbalance to SCM, including abnormal copper metabolism (dysregulated uptake/transport/excretion), lipid metabolism disorders, endoplasmic reticulum stress (ERS), and various regulated cell death (RCD) forms (cuproptosis, apoptosis, autophagy, pyroptosis, ferroptosis, necrosis). We also elaborate potential therapeutic strategies targeting copper homeostasis, including copper chelators, copper transport inhibitors, copper-mediated RCD modulators, multi-target natural products, nanopreparations, and latest advances in copper-based myocardial injury therapy. Finally, we address current research limitations and outline future directions, such as exploring copper-related cell death markers, clarifying underexplored copper signaling in SCM, and developing innovative precision therapies. This review offers a comprehensive theoretical foundation for further investigating copper homeostasis in SCM and developing novel therapies.

## 1 Introduction

Sepsis is a life-threatening clinical syndrome triggered by the invasion of pathogenic microorganisms (e.g., bacteria, fungi, viruses, and parasites) into sterile human tissues, which induces a dysregulated host response to infection and culminates in critical organ dysfunction. Ranked among the top ten causes of mortality globally in both developed and developing nations, sepsis imposes a substantial public health burden (Hawiger, 2018). Sepsis-induced cardiomyopathy (SCM), a reversible cardiac dysfunction associated with sepsis, is primarily characterized by impaired left ventricular systolic function. Frequently concurrent with septic shock, SCM manifests as functional and structural cardiac damage driven by the release of inflammatory mediators, ultimately leading to cardiomyocyte injury and subsequent deterioration of cardiac function (Sato and Nasu, 2015).

The pathogenesis of SCM involves multifaceted and interconnected mechanisms, including inflammatory mediators (Song et al., 2023a), oxidative stress, mitochondrial dysfunction (Hollenberg and Singer, 2021), dysregulated autophagy (Fan and Wu, 2024), immune responses, pathogen-associated molecular patterns (PAMPs), damage-associated molecular patterns (DAMPs) (Kakihana et al., 2016), and hemodynamic perturbations (Fan and Wu, 2024). Reported incidence rates of SCM vary substantially (10%–70%) across studies, likely due to heterogeneity in diagnostic criteria, study designs, and confounding factors. Current therapeutic strategies prioritize early fluid resuscitation, inotropic agents, and vasopressors to control infections and optimize hemodynamics. Elucidating the molecular mechanisms underlying SCM and developing targeted therapeutic interventions remain urgent priorities in critical care medicine (Salami et al., 2024). The onset of SCM has been associated with multiple factors, one of which is an copper dyshomeostasis.

Copper (Cu) is a crucial catalytic cofactor in various physiological processes in the human body, including energy metabolism, immunomodulation, and antioxidant activity (Chen et al., 2020). It exists in two primary oxidized forms, Cu (II) and Cu (I), within different cellular compartments. This trace element plays an indispensable role in biological processes such as mitochondrial respiration, antioxidant defenses, and biocompound synthesis (Han, 2023). Tight regulation of Cu concentration is essential to prevent imbalances that may induce cell death (Yang L. et al., 2023).

The mechanisms by which copper contributes to disease pathogenesis form an intricate network, centrally involving copper dyshomeostasis and its consequences, including oxidative stress (Golec et al., 2021), mitochondrial damage (Rodriguez et al., 2024), protein aggregation (Zou et al., 2021), immune escape (Yi et al., 2022), angiogenesis (Gérard et al., 2010), and inflammatory modulation (Tao et al., 2021). Specifically, copper catalyzes the Fenton reaction, generating reactive oxygen species (ROS) that directly cause oxidative damage to critical biomacromolecules such as lipids, proteins, and DNA (Golec et al., 2021). Concurrently, copper accumulates within mitochondria, where it impairs the function of respiratory chain complexes. This leads to reduced ATP synthesis and directly disrupts the mitochondrial membrane potential, and thereby induces the opening of the mitochondrial permeability transition pore (mPTP) and the subsequent release of pro-apoptotic factors (Sebők-Nagy et al., 2023; Zheng et al., 2023).

**FIGURE 1:**
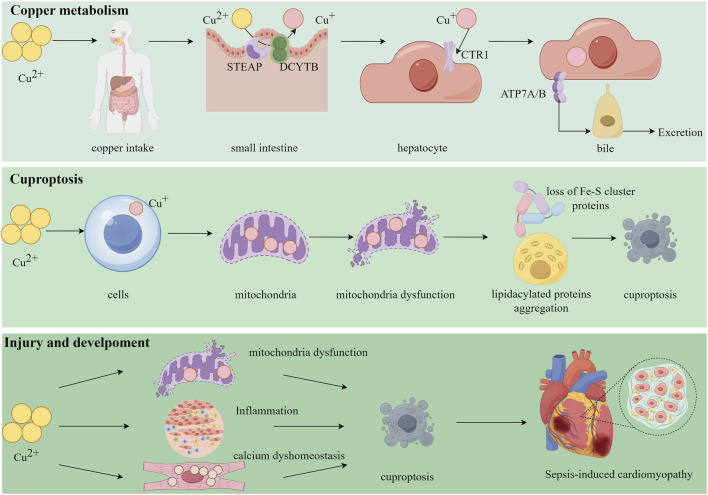
Copper plays a critical role in cellular processes and disease pathogenesis. Following uptake into the body, Cu^2+^ is reduced to Cu^+^ by epithelial antigen of the prostate (STEAP) and duodenal cytochrome b (DCYBT), and subsequently imported into hepatocytes via the copper transporter 1 (CTR1). The intracellular copper is then exported into bile for elimination by the transporters copper-transporting ATPase alpha/beta (ATP7A/B). Excess copper disrupts mitochondrial function, leading to the loss of Fe-S cluster proteins and the aggregation of lipoylated proteins, which ultimately triggers cuproptosis, a novel form of regulated cell death. In the context of sepsis-induced cardiomyopathy, excessive copper promotes mitochondrial damage, inflammatory infiltration, and disruption of calcium homeostasis in cardiomyocytes, culminating in cuproptosis and the development of cardiac dysfunction.

Furthermore, copper also modulates disease progression by regulating inflammatory signaling pathways and immune cell functions. In promoting angiogenesis, copper activates multiple pro-angiogenic and inflammatory factors; it also directly binds to angiopoietin (ANG), modulating its affinity for endothelial cells to facilitate blood vessel formation (Giacomelli et al., 2015). In summary, these multifaceted and interconnected mechanisms form the basis of copper’s role in disease and offer novel avenues for therapeutic strategies targeting copper balance (Figure 1).

This review delves into the current literature on copper homeostasis in SCM, examining the pathological processes arising from disruptions in copper balance. It thoroughly explores the correlation between copper homeostasis and the molecular mechanisms underlying SCM development. Moreover, it introduces novel perspectives on utilizing copper homeostasis for SCM treatments and proposes potential directions for addressing copper dyshomeostasis in the future.

## 2 Copper homeostasis biochemical and molecular insights

### 2.1 Copper uptake and transport

Copper is an essential cofactor widely distributed in nature and obtained by the human body through dietary intake. Dietary copper, primarily exists in the form of Cu^2+^ (copper ion, oxidized form), but only Cu^+^ (cuprous ion, reduced form) can be absorbed and reused; it is decomposed and absorbed mainly in the duodenum and small intestine (Mason, 1979). This absorption process is aided by metal reductases such as Six-transmembrane Epithelial Antigen of the Prostate and Duodenal Cytochrome b (DCYTB).

Cu^+^ is taken up into the cell and conveyed by copper transporters 1 (CTR1) on the apical side of the enterocyte (Zhou et al., 2023), then transported to the trans Golgi Network (TGN) by antioxidant 1 (ATOX1). Copper-transporting ATPase alpha (ATP7A) carries copper from the Golgi apparatus to the basolateral region of the cell, from which it is released into the portal circulation. The liver acquires copper from the portal circulation, and copper-transporting ATPase beta (ATP7B) utilizes it to metallate newly synthesized ceruloplasmin (CP); the resulting copper-containing proteins are subsequently distributed to peripheral organs via the bloodstream (Kahra et al., 2015; Petruzzelli and Polishchuk, 2019).

The liver acts as the primary organ for copper storage and excretion, playing a central role in the regulation of systemic copper homeostasis. Within hepatocytes, copper is facilitated for storage by binding to metallothioneins (MTs). MTs are thiol-rich, reducing molecules that exhibit high affinity for copper and are essential for maintaining copper equilibrium by storing and releasing excess copper in a regulated manner (Tsvetkov et al., 2022; Xie et al., 2023).

Copper trafficking is tightly controlled by a series of copper chaperones. For instance, mitochondria contain cytochrome c oxidase copper chaperone 17 (COX17), which delivers Cu^+^ to secondary copper carrier proteins like cytochrome c oxidase subunit 1 (COX1) and cytochrome c oxidase subunit 2 (COX2). Additionally, the copper chaperone for superoxide dismutase (CCS) serves a critical function in delivering Cu^+^ to subunit 1 of superoxide dismutase 1 (SOD1) to support its antioxidative stress activity (La Fontaine et al., 2010).

### 2.2 Copper storage and export

Copper is not sequestered in a single centralized tissue but is widely distributed throughout various organs, with the liver serving as the central hub for systemic copper metabolism and storage (Camargo et al., 2023; Murillo et al., 2022). Subsequent to intestinal absorption, copper is released into the systemic circulation and associates with soluble chaperones including CP, human serum albumin, macroglobulin, and histidine (Lutsenko, 2021; Moriya et al., 2008; Ramos et al., 2016). These complexes transport copper to the liver through the portal vein, where hepatocytes primarilyt ake it up via CTR1. The liver then incorporates copper into proteins, such as ceruloplasmin, sequesters it in MTs, and releases it back into circulation or secretes it into bile in response to systemic demands. Thus, the liver acts as the primary organ for copper capture, distribution, and elimination, playing a pivotal role in maintaining systemic copper homeostasis.

**FIGURE 2:**
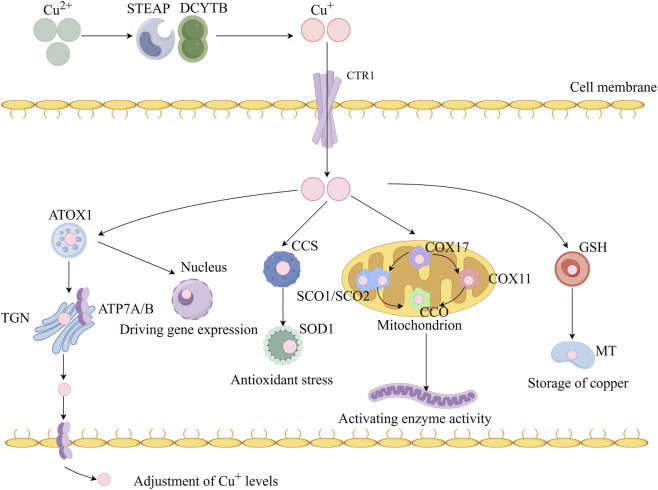
Copper metabolism at the cellular level. Extracellular Cu^2+^ is reduced by the reductase six-transmembrane epithelial antigen of the prostate (STEAP) and duodenal cytochrome b (DCYBT) to Cu^+^, which is transported into the cell by the Cu transporter copper transporter 1 (CTR1), where it is delivered to cytosolic Cu chaperones such as copper chaperone for superoxide dismutase (CCS) and superoxide dismutase 1 (SOD1) and then delivered to specific subcellular compartments such as the mitochondria, trans Golgi network (TGN), and nucleus. Antioxidant-1 (ATOX1) transports Cu^+^ to the nucleus, where it binds to transcription factors and drives gene expression, In the nucleus, Cu can bind to transcription factors and drive gene expression, In the TGN, copper-transporting ATPase alpha (ATP7A) and copper-transporting ATPase beta (ATP7B), which are Cu^+^-ATPase transporters, transfer Cu to its lumen, When intracellular Cu + increases, these Cu^+^-ATPase fuse with the plasma membrane to export Cu^+^. In the basolateral membrane of enterocytes, ATP7A facilitates the pumping of copper into the portal circulation, where it subsequently enters the liver, the primary organ responsible for copper storage. In the mitochondria, cytochrome C oxidase copper chaperone 17 (COX17) transports Cu^+^ to the copper-carrying proteins synthesis of cytochrome C oxidase 1 (SCO1), synthesis of cytochrome C oxidase 2 (SCO2), and cytochrome c oxidase copper chaperone 11 (COX11) and delivers it to cytochrome C oxidase (COX) to activate the activity of enzymes in the respiratory chain. Cu can be sequestered by metallothionein (MT) for storage.

Biliary excretion repreents the major pathway for copper elimination from the body (Linder, 2020), through which excess copper is secreted by hepatocytes into bile and ultimately eliminated in feces (Wijmenga and Klomp, 2004). ATP7A and ATP7B mediate copper transport in peripheral tissues and the liver, respectively (Telianidis et al., 2013). Specifically, ATP7A facilitates copper delivery into the portal circulation, whereas ATP7B pumps copper from the liver back into the systemic circulation. Mutations in ATP7A and ATP7B can lead to copper metabolism disorders, such as Menkes disease and Wilson’s disease (Horn and Wittung-Stafshede, 2021; Schushan et al., 2012) (Figure 2).

### 2.3 Copper homeostasis and its physiological functions in the cardiac system

The core physiological roles of copper in the heart are primarily exerted through its function as a cofactor for several key enzymes, which are involved in fundamental biological processes such as energy metabolism, antioxidant defense, and connective tissue formation (James, 2021). Copper is an essential component of COX, an enzyme localized to the inner mitochondrial membrane. As the terminal complex of the oxidative phosphorylation pathway, COX is responsible for shuttling electrons to oxygen to form water while driving ATP synthesis. Thus, copper is vital for the energy supply of cardiomyocytes, which exhibit high metabolic demands.

Furthermore, copper acts as a critical prosthetic group for SOD, specifically the SOD1. This enzyme converts harmful superoxide anion radicals generated during cardiac metabolism into hydrogen peroxide, representing the primary defense against oxidative stress and protecting cardiomyocytes from oxidative damage. Additionally, copper serves as a necessary cofactor for lysyl oxidase, which catalyzes the formation of cross-links in collagen and elastin. This function is crucial for maintaining the strength and elasticity of cardiac connective tissues, such as heart valves and blood vessel walls (Lawler and Song, 2002).

### 2.4 Disruption of copper homeostasis by sepsis

Sepsis is a systemic inflammatory response syndrome triggered by pathogen invasion, which can lead to multiple organ dysfunction. Accumulating evidence suggests that severe infection consistently disrupts the homeostasis of metal ions (Zhang et al., 2025). CP is classified as an acute-phase protein, and its synthesis is significantly upregulated under inflammatory stimulation. This prompts the liver to incorporate more copper into CP and release it into the bloodstream, potentially resulting in elevated serum copper levels while perturbing copper distribution in tissues (Liu Z. et al., 2022).

Furthermore, pathogen invasion and systemic inflammation induce massive overproduction of ROS and nitrogen species. These molecules, particularly nitric oxide and its derivatives such as peroxynitrite, have been demonstrated to directly inhibit the activity of key antioxidant enzymes, including SOD1. This impairment compromises the heart’s antioxidant defense system, exacerbating a vicious cycle between copper dyshomeostasis and oxidative stress. Under conditions of severe oxidative stress, ROS and nitrogen species can target copper-containing proteins, including the dissociation of the copper cofactor from their active sites and leading to the release of free copper ions (Lawler and Song, 2002). These free copper ions can then catalyze the generation of more destructive hydroxyl radicals via the Fenton reaction, resulting in lipid peroxidation, protein denaturation, and DNA damage, which directly injures functional structures of cardiomyocytes such as cell membranes and mitochondria (Jomova et al., 2022).

The pathological manifestations of cardiac copper dyshomeostasis are diverse and mechanistically complex. Copper deficiency primarily leads to myocardial hypertrophy, remodeling, and alterations in systolic and diastolic function. Its core mechanisms involve calcium dysregulation due to SERCA2a dysfunction and the inhibition of autophagy. In contrast, copper overload is closely associated with heart failure, particularly with reduced systolic function, likely through pathways that induce cuproptosis, exacerbate oxidative stress, and interact with ferroptosis mechanisms, thereby causing myocardial damage.

## 3 Pathogenic mechanisms of copper dyshomeostasis in SCM

### 3.1 Core pathological processes of copper dyshomeostasis in SCM

SCM is an acute cardiac dysfunction syndrome triggered by systemic infection and inflammation. If it is untreated, it can progress to multiple organ failure, underscoring the critical importance of prompt infection source control and immune function enhancement in SCM management. Emerging research underscores the role of copper dyshomeostasis—characterized by pathological copper excess or deficiency—as a key contributor to SCM pathogenesis, highlighting its potential as a therapeutic target.

Accumulating evidence supports a significant association between elevated systemic copper levels and cardiovascular disease. Large-scale prospective cohort studies indicate that increased serum copper concentrations correlate with higher mortality risk in cardiovascular diseases, particularly coronary heart disease (Ford, 2000; Kok et al., 1988; Reunanen et al., 1996). Furthermore, a study by Jaakko T. Laine et al. establishes a link between elevated copper-to-zinc (Cu/Zn) ratios—predominantly driven by increased serum copper levels—and heightened infection susceptibility in middle-aged and elderly populations (Laine et al., 2020). A single-center prospective observational study of patients with septic shock investigated the relationship between cardiac dysfunction and whole blood levels of Cu, Zn, and the Cu/Zn ratio. Participants were divided into three groups: septic shock with sepsis-induced left ventricular systolic dysfunction (SILVSD), septic shock without myocardial dysfunction, and healthy controls. The results demonstrated that compared with both the non-dysfunction septic shock group and the healthy control group, patients in the SILVSD group exhibited significantly elevated whole blood copper levels and a higher Cu/Zn ratio, along with significantly reduced Zn levels. Collectively, these findings implicate copper dyshomeostasis in SCM pathogenesis and underscore the critical importance of maintaining systemic copper homeostasis.

Inflammation, a key clinical feature of SCM, has been mechanistically linked to copper homeostasis in recent studies. As an essential trace element, copper plays a critical role in human and animal physiology. While copper deficiency impairs immune function and elevates susceptibility to infections and inflammatory disorders; excessive copper accumulation induces cellular and organ damage, exacerbating inflammatory responses (Deng et al., 2023).

Emerging evidence illustrates the dual roles of copper in inflammatory processes: in conditions such as rheumatoid arthritis (RA), systemic inflammation elevates serum copper levels and enhances synthesis of copper-binding proteins like CP, thereby increasing copper availability and conferring anti-inflammatory effects (Zhang W. et al., 2025). Conversely, elevated copper levels stimulate the production of pro-inflammatory cytokines, including interleukin-1β (IL-1β) and tumor necrosis factor-α (TNF-α) (Jian et al., 2020). This reciprocal interplay suggests a self-reinforcing cycle between copper dysregulation and inflammatory activation. Furthermore, copper-mediated oxidative stress perpetuates inflammation through excessive ROS accumulation, which enhances myeloperoxidase activity, activates the nuclear factor-κB (NF-κB) pathway, downregulates anti-inflammatory cytokines, and promotes a proinflammatory microenvironment (Petris and Mercer, 1999).

Mitochondria generate ATP through oxidative phosphorylation, serving as the primary energy source for organisms. These organelles harbor cytochrome c oxidase (COX), a critical component of biological function (Garza et al., 2023). In sepsis, mitochondrial function in cardiomyocytes is impaired, manifesting as morphological alterations and functional deficits. Such damage reduces the efficiency of oxidative phosphorylation, thereby diminishing ATP production and compromising myocardial contractility (Durand et al., 2017; Tan et al., 2019). Elevated levels of copper ions promote the generation of ROS via the Fenton reaction, thereby exacerbating oxidative stress and damaging the mitochondrial membrane potential. Furthermore, ROS can induce additional loss of membrane potential by activating the mPTP, ultimately triggering cell apoptosis. Mitochondria actively sequester large quantities of calcium ions, a process essential for maintaining intracellular calcium homeostasis and ensuring cellular survival and function (Pivovarova and Andrews, 2010). Beyond their role in energy metabolism, mitochondria are key sites for copper storage and dynamic regulation. Copper enters mitochondria via copper transporters (e.g., CTR1) to modulate the activity of respiratory chain enzymes such as COX (Oliveri, 2020; Tian et al., 2023). While copper serves as an indispensable cofactor for mitochondrial COX, excessive copper accumulation damages cellular organelles. Stable copper levels are prerequisite for sustaining normal mitochondrial respiratory function. Copper overload impairs mitochondrial function through multiple pathological pathways (Zischka and Einer, 2018), including Fenton reaction-driven ROS generation, which induces DNA damage and lipid peroxidation. Additionally, copper disrupts the ubiquitin-proteasome system and inhibits cellular protease activity by interfering with cell proliferation. Furthermore, copper binding to lipidated proteins promotes their aggregation, ultimately triggering cell death (Liu et al., 2023; Ruiz et al., 2021).

Cardiac dysfunction in SCM, characterized by impaired systolic and diastolic function, is centrally linked to dysregulated calcium (Ca^2+^) homeostasis. Proinflammatory cytokines and ROS inhibit the sarcoplasmic reticulum Ca^2+^-ATPase (SERCA2), compromising cytosolic Ca^2+^ reuptake and leading to diastolic dysfunction and calcium overload (Bertero and Maack, 2018; Gonnot et al., 2023; Hobai et al., 2013). Concurrently, sepsis-induced copper dyshomeostasis may impair the activity of copper-dependent antioxidant enzymes like SOD, exacerbating ROS accumulation (Gaetke and Chow, 2003). This excess ROS further disrupts Ca^2+^ handling (De Nicolo et al., 2023; Zhou et al., 2019), creating a vicious cycle. The ensuing Ca^2+^ dysregulation can trigger pathological opening of the mitochondrial permeability transition pore (mPTP), resulting in mitochondrial Ca^2+^ overload, swelling, diminished ATP synthesis, and activation of apoptotic pathways (Halestrap et al., 2004). Thus, copper imbalance likely perturbs Ca^2+^ homeostasis indirectly by amplifying oxidative stress, mitochondrial dysfunction, and inflammation, which in turn exacerbates cellular injury—forming a deleterious positive feedback loop. Current evidence predominantly focuses on isolated ion mechanisms, and the direct role of copper in this context requires further experimental validation.

**FIGURE 3:**
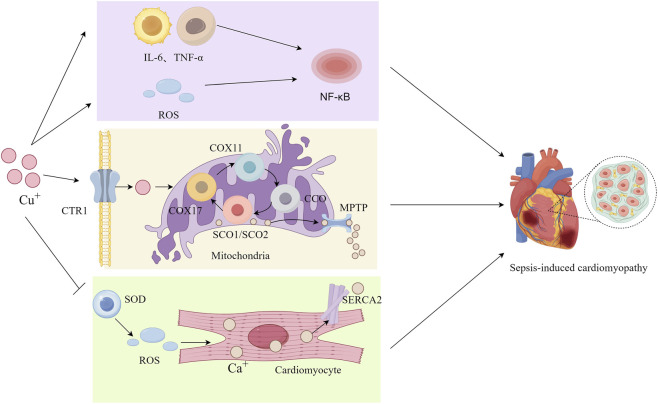
Copper in Septic-induced Cardiomyopathy. Excessive copper promote the production of inflammatory factors (e.g., IL-1β and TNF-α) and reactive oxygen species (ROS), thereby activating the nuclear factor-κB (NF-κB) signaling pathway. The influx of excess copper into cells via copper transporter 1 (CTR1) induces mitochondrial damage. Within cardiomyocytes, elevated copper levels inhibit superoxide dismutase (SOD) activity and exacerbate ROS accumulation. Concurrently, copper suppresses the expression of sarcoplasmic reticulum Ca^2+^-ATPase (SERCA2), thereby reducing calcium uptake by the sarcoplasmic reticulum and leading to disruption of calcium homeostasis. Furthermore, this calcium dysregulation triggers aberrant opening of the mitochondrial permeability transition pore (MPTP), which exacerbates mitochondrial injury. Collectively, these cascade events ultimately contribute to the pathogenesis of septic cardiomyopathy.

However, the pathogenesis of SCM is complex and multifaceted. Current understanding is largely inferred from pathophysiological alterations observed during SCM onset, which suggest a potential interplay between copper dysregulation and disease progression. Nevertheless, emerging evidence underscores the pivotal role of copper homeostasis in both vascular disorders and SCM pathogenesis, highlighting its potential as a critical therapeutic target in mitigating systemic inflammation and cardiac dysfunction (Figure 3).

### 3.2 Mechanistic insights and emerging evidence linking copper dyshomeostasis to SCM

#### 3.2.1 Oxidative stress

Oxidative stress plays a critical role in SCM (Zhang et al., 2022). Oxidative stress is characterized by an imbalance between cellular ROS generation and endogenous antioxidant defense capacity. ROS are constitutively produced as metabolic byproducts of mitochondrial respiration and other enzymatic reactions. In the myocardium, major sources of ROS include the mitochondrial electron transport chain, xanthine oxidase, NADPH oxidase (NOX), and nitric oxide (NO) synthase. Disturbances in redox homeostasis within the cardiovascular system directly promote oxidative stress (Dubois-Deruy et al., 2020).

Copper may exacerbate oxidative stress by stimulating ROS production and participating in the Fenton reaction. Copper cycle between oxidized and reduced states, thereby generating highly reactive hydroxyl radicals (Husain and Mahmood, 2019). These hydroxyl radicals induce lipid peroxidation, protein oxidation, and DNA damage, ultimately contributing to the initiation and progression of atherosclerosis (Blades et al., 2021).

Moreover, copper deficiency may also augment mitochondrial oxidative stress by suppressing COX (complex IV) activity, which impairs electron transport chain function and secondarily increases ROS formation (Johnson and Thomas, 1999).

#### 3.2.2 Lipid metabolism

Accumulating evidence indicates that copper modulates lipid metabolism in the human body. Copper deficiency can result in elevated serum lipid levels, while copper excess may reduce the expression of ATP7B and serum cholesterol concertrations. The regulatory role of Cu in lipid metabolism is multifaceted and complex. Cu is involved in the oxidative metabolism of fatty acids, promoting their catabolism and metabolism. The liver plays a significant role in coordinating copper homeostasis and lipid metabolism (Morrell et al., 2017).

Excess Cu in hepatocytes can inhibit cholesterol biosynthesis, leading to decreased cholesterol levels in both the liver and serum, thereby exerting a detrimental effect on lipid metabolic balance. In contrast, Cu deficiency is associated with increased levels of both high-density lipoprotein (HDL) and low-density lipoprotein (LDL), which in turn result in elevated systemic levels of cholesterol, triglycerides, and phospholipids (Blades et al., 2021).

ATP7A serves as the primary exporter of Cu in enterocytes, facilitating Cu release into the bloodstream for systemic distribution. Deficiencies in ATP7A have been shown to increase the accumulation of Cu within enterocytes. Meanwhile, ATP7B functions as a Cu reservoir in the intestine tissues, and impairments in its activity can reduce the absorption of dietary fats (Guttmann et al., 2020).

Collectively, Cu deficiency is associated with increased adipocyte formation, reduced lipid transport, and impaired lipid breakdown, ultimately resulting in enhanced lipid accumulation in the body. Conversely, copper excess is associated with decreased adipocyte differentiation and increased lipid catabolism, leading to an overall reduction in systemic lipid levels. Additionally, copper participates in the regulation of antioxidant enzyme activity, directly influencing lipid accumulation and oxidative stress; it can also induce lipid deposition through promoting oxidative stress and mitochondrial dysfunction.

Research has revealed that abnormal accumulation and decreased polarity of lipid droplets (LDs) within cardiomyocytes are important molecular features of SCM (Zhang X. et al., 2024). LDs are crucial subcellular organelles for the storage and metabolism of lipids, and changes in their polarity directly reflect alterations in the lipid metabolic microenvironment. Although existing literature has not directly investigated the relationship between copper homeostasis and lipid metabolism, based on the well-established biological foundation of copper as an essential cofactor for core enzymes involved in lipid metabolism, oxidative stress, and energy production, it is reasonable to infer that copper dyshomeostasis likely contributes to the pathological progression of SCM by affecting lipid metabolism.

#### 3.2.3 Endoplasmic reticulum stress

The endoplasmic reticulum (ER) is a key organelle in eukaryotic cells, essential for protein synthesis, folding, transport, and intracellular calcium storage. Factors like genetic diseases and external environment can trigger the production of mutant proteins and disrupt the redox balance, leading to the unfolded protein response (UPR). When the ER’s folding capacity is overwhelmed by the volume of secreted proteins, misfolded proteins accumulate, resulting in endoplasmic reticulum stress (ERS) (Guo et al., 2020).

Recent studies have suggested a potential link between copper homeostasis, ERS, and cardiovascular disease (CVD). Evidence indicates that elevated Cu ion levels can exacerbate ER damage, while inhibiting ERS can reduce cellular apoptosis, implying that Cu may regulate ERS and apoptotic processes (Gu et al., 2025; Ma et al., 2023). Furthermore, excessive copper accumulation can induce ROS production, which further initiates oxidative stress and impairs ER function (Xue et al., 2023a).

Oxidative stress disrupts ER membrane stability, leading to impairments in protein folding. Additionally, excessive copper can disturb intracellular calcium ion homeostasis, which is critical for noemal ER function. Alterations in calcium ion concentration affect protein folding and transport, subsequently inducing ERS (Groenendyk et al., 2014). When copper concentration exceeds physiological levels, it triggers cytotoxicity and a unique form of cell death termed cuproptosis. Thus, maintaining stable copper ion levels within cellular compartments is extremely critical (Chen et al., 2022).

Furthermore, excessive copper intake has been linked to oxidative stress induction, which in turn promotes both ERS and mitochondrial pathway impairment, ultimately leading to cardiomyocyte apoptosis (Li Q. et al., 2022).

#### 3.2.4 Regulatory cell death

Regulatory cell death (RCD), also known as programmed cell death, is a highly regulated and controlled process that occurs in multicellular organisms. It holds profound physiological and pathological significance in maintaining the stability of the organism’s internal environment and promoting normal growth and development. RCD encompasses various forms of cell death, including apoptosis, autophagy, pyroptosis, ferroptosis, and necrosis (Gao and Zhang, 2023).

Cu is a crucial component of several enzymes, such as SOD, COX, and tyrosinase, which play vital roles in the body’s electron transport process. Imbalance in copper homeostasis, whether caused by Cu overload or deficiency, can lead to abnormal cellular function and ultimately result in cellular death.

Recent studies have identified a novel mode of RCD termed cuproptosis. This process involves intracellular copper binding to lipidated constituents in the tricarboxylic acid (TCA) cycle. The aggregation of these copper-bound lipidated mitochondrial proteins, as well as the subsequent reduction of iron-sulfur (Fe-S) clusters, induces proteotoxic stress that ultimately leads to cellular death (Tsvetkov et al., 2022). Thus, maintaining stable copper ion levels within cellular compartments is extremely critical for preventing abnormal RCD and protecting cardiomyocyte function.

##### 3.2.4.1 Apoptosis

Apoptosis is a classic form of programmed cell death mediated by a cascade of caspase-family proteases. It is characterized by cell shrinkage, nuclear condensation and fragmentation, the formation of dynamic membrane blebs, and the proteolytic cleavage of intracellular substrates (Hu et al., 2022). Although the mechanisms underlying apoptosis and cuproptosis are distinct, they can exhibit significant synergistic effects. Within the cell, copper function as a double-edged sword. On one hand, they serve as essential cofactors for numerous enzymes, thereby supporting cellular growth. On the other hand, copper at supraphysiological concentrations can induce oxidative damage and apoptosis through ROS generation via the Fenton reaction, while simultaneously triggering cuproptosis by targeting mitochondrial proteins (He et al., 2024; Li et al., 2024; Song et al., 2023b). Consequently, copper act as a pivotal link bridging these two cell death pathways.

As previously mentioned, copper is a crucial factor in the functional regulation and stability maintenance of various enzymes, some of which are involved in apoptotic pathways. For instance, COX is paramount for maintaining mitochondrial function; impairment of COX can lead to mitochondrial dysfunction, alterations in mitochondrial membrane permeability, and subsequent mitochondrial dysregulation. Copper can also damage the mitochondrial electron transport chain and inhibit ATP synthesis, further exacerbating cellular energy metabolism disorders, potentially triggering the activation of apoptotic signaling pathways and culminating in cell death (Ruiz et al., 2021).

Cuproptosis and apoptosis represent two distinct forms of RCD. Apoptosis is a finely genetically programmed “routine” cellular clearance process, whereas cuproptosis is a “metabolic” cell death event directly driven by copper ion toxicity. The former relies on the caspase-mediated dismantling of cellular systems, while the latter depends on the disruptive aggregation of mitochondrial metabolic proteins by copper ions. This fundamental distinction not only provides a novel perspective for understanding the pathophysiology of various diseases but also opens up an entirely new therapeutic domain.

##### 3.2.4.2 Autophagy

Copper can trigger autophagy (Tsang et al., 2020). Autophagy, a catabolic recycling pathway, plays a crucial role in cellular survival under various stress conditions and is of great significance in CVD (Rabinovich-Nikitin et al., 2023). There are three main types of autophagy: macroautophagy, microautophagy, and chaperone-mediated autophagy (CMA) (Gatica et al., 2022). Among them, macroautophagy, the primary branch, is particularly involved in cardiovascular pathophysiology.

The macroautophagy process involves the formation of autophagosomes around damaged cellular components, fusion with lysosomes to form autophagolysosomes, and subsequent degradation of encapsulated contents by lysosomal enzymes. AMP-activated protein kinase (AMPK) acts as an energy sensor during this process, phosphorylating key proteins to initiate autophagy. Key steps of macroautophagy include vesicle formation and fusion, recruitment of autophagy-related proteins (ATG), and membrane elongation. Eventually, autophagosomes fuse with lysosomes to form autophagolysosomes, leading to the production of catabolic metabolites for cellular energy metabolism (Rabinovich-Nikitin et al., 2023).

Cu can modulate the autophagic process *in vivo* through multiple mechanisms. It can trigger autophagy by upregulating ATG expression and modulating the AMPK-mTOR pathway, or inducing oxidative stress (Zhong et al., 2022). ATG-5, a critical protein, binds to other autophagic proteins to form a complex that facilitates autophagosome formation (Wan et al., 2020). The AMPK-mTOR pathway plays a vital role in autophagy signaling. Autophagy, regulated by AMPK, serves as a key sensor in controlling cellular metabolism and maintaining energy balance. Activation of AMPK can induce autophagy by inhibiting mTOR (Guo et al., 2022).

Notably, Dihydrolipoamide S-acetyltransferase (DLAT) functions not only as a key executor of cuproptosis but also as a modulator of autophagy. In hepatocellular carcinoma, knockdown of DLAT has been shown to suppress autophagy and induce cell death (Yang Q. et al., 2023). In contrast, in prostate cancer, cuproptosis-induced upregulation of DLAT inhibits autophagy through the mTOR pathway (Wen et al., 2023). These findings highlight the pivotal role of DLAT as a central regulatory node linking cuproptosis and autophagy. Moreover, deletion of the ATP7B can activate the transcription factor EB (TFEB), thereby promoting autophagy (Xue et al., 2023a).

The impact of autophagy on cuproptosis is not unidirectional but exhibits a dual regulatory role, capable of either promoting or inhibiting the process. Under certain conditions, inhibition of autophagy enhances cuproptosis. For instance, in an achondroplasia model, mutation of fibroblast growth factor receptor 3 (FGFR3) suppresses autophagy in chondrocytes, thereby promoting Heat shock protein B 6 (HSPB6)-mediated cuproptosis (Chen et al., 2025). Conversely, in other contexts, activation of autophagy may counteract cuproptosis. In a periodontitis model, the copper chelator tetrathiomolybdate (TTM) alleviated inflammation by inhibiting cuproptosis while simultaneously enhancing autophagic flux (Zhang L. et al., 2024).

In summary, copper dyshomeostasis is a significant potential driver in the pathogenesis of SCM. As a key cofactor for numerous core enzymes, disruption of copper homeostasis directly interferes with cellular autophagy. In the setting of sepsis, copper dyshomeostasis may impair autophagosome formation or their fusion with lysosomes, leading to organelle damage, such as dysfunctional mitochondria (Kim et al., 2021). This impairment of autophagic flux exacerbates oxidative stress and energy crisis in cardiomyocytes on one hand (Xu et al., 2024), and hinders the normal turnover of lipid droplets on the other, further worsening pre-existing lipid metabolic disturbances within the cells (An et al., 2017; Ou et al., 2025; Singh et al., 2009). Consequently, a vicious cycle of mutual exacerbation is formed among copper dyshomeostasis, autophagic dysfunction, myocardial lipotoxicity, and mitochondrial injury, which collectively drives the pathological progression of cardiomyocyte damage and contractile dysfunction in SCM.

##### 3.2.4.3 Pyroptosis

The core mechanism of pyroptosis involves gasdermin D (GSDMD)-mediated membrane pore formation, leading to cell swelling, rapid lysis, and the massive release of pro-inflammatory mediators such as interleukin-1β (IL-1β) and interleukin-18 (IL-18) (Wang et al., 2020). The burst of ROS serves as a critical link connecting pyroptosis and cuproptosis. Many copper-based nanomaterials can generate substantial amounts of ROS in the tumor microenvironment through pathways such as Fenton-like reactions (Cun et al., 2025; Zhong et al., 2024).

On one hand, these ROS can directly or indirectly activate the NLRP3 inflammasome/Caspase-1 pathway, resulting in GSDMD cleavage and the induction of pyroptosis. On the other hand, ROS-induced oxidative stress can impair mitochondrial function and downregulate the expression of the copper transporter ATP7A, thereby reducing cellular copper export. This subsequently leads to intracellular copper accumulation and promotes cuproptosis (Zhao et al., 2023).

Notably, mitochondria, the primary site of cuproptosis, are also closely involved in regulating pyroptosis. During cuproptosis, the aggregation and functional collapse of mitochondrial lipoylated proteins directly induce severe mitochondrial stress. This stress can trigger events such as mitochondrial membrane potential collapse, massive ROS production, and COX release. These signals can further activate inflammasomes or the Caspase-3/GSDME pathway, thereby initiating or amplifying pyroptosis (Liu et al., 2024; Zhu et al., 2024). Consequently, mitochondria act as a pivotal hub for transmitting cuproptosis signals to pyroptosis pathways.

In summary, while cuproptosis is centered on metabolic toxicity, pyroptosis focuses on inflammatory signaling. Their most significant relationship lies in serial amplification: as a severe form of mitochondrial damage, cuproptosis acts as a potent upstream signal that activates the classical inflammasome-pyroptosis pathway, thereby transforming a metabolic cell death event into a robust inflammatory response.

##### 3.2.4.4 Ferroptosis

Previous studies have demonstrated that there is another form of cell death associated with-ferroptosis (Jiang et al., 2021). This type of cell death is characterized by the generation of lipid ROS and disruption of iron homeostasis. Ren et al. found that disulfiram/copper treatment of hepatocellular carcinoma (HCC) cells disrupted mitochondrial homeostasis, resulting in mitochondrial fragmentation and accumulation around the nucleus in HCC cells. Additionally, this treatment induced superoxide accumulation, lipid peroxidation, and an increase in the free iron pool, suggesting that copper induces both cuproptosis and ferroptosis (Ren et al., 2021).

Glutathione peroxidase 4 (GPX4) is a central regulator of ferroptosis. Copper directly binds to GPX4, inducing its aggregation and subsequent autophagic degradation, which ultimately promotes ferroptotic cell death (Xue et al., 2023b). Copper chelators can selectively attenuate cellular susceptibility to ferroptosis without significantly influencing other forms of cell death. In contrast, the natural flavonoid fisetin exerts cardioprotective effects by upregulating GPX4 expression, thereby enhancing its antioxidant capacity, inhibiting ferroptosis, and ameliorating cardiac hypertrophy (Li D. et al., 2022).

Furthermore, copper-binding agents and their corresponding copper complexes—such as elesclomol-copper and disulfiram-copper—disrupt mitochondrial homeostasis and induce oxidative stress, ultimately triggering ferroptosis in cancer cells (Arkan and Akcora-Yildiz, 2025; Gao et al., 2024; Wang et al., 2024; Zhao and Zhu, 2024). These findings are consistent with prior evidence that excessive copper accumulation elevates intracellular ROS levels in cardiomyocytes.

Cuproptosis and ferroptosis are two distinct forms of metal ion-dependent regulated cell death. Cuproptosis is driven by excessive copper ions, with its core mechanism involving the direct binding of copper to lipoylated proteins in the mitochondrial TCA cycle, leading to abnormal aggregation and functional impairment of these proteins. This subsequently triggers proteotoxic stress and ultimately disrupts cellular metabolism. In contrast, ferroptosis depends on iron ions and is characterized by the failure to repair lipid peroxides due to the loss of GPX4 activity, resulting in irreversible membrane damage, primarily featuring the peroxidation of polyunsaturated fatty acids. In brief, cuproptosis is a “proteotoxic catastrophe” induced by copper in mitochondrial metabolism, whereas ferroptosis is a “membrane integrity collapse” caused by iron-catalyzed lipid peroxidation.

##### 3.2.4.5 Necrosis

Necrosis has traditionally been regarded as a non-programmed, passive form of cell death, typically triggered by severe physical or chemical insults. It is characterized by cell swelling, loss of membrane integrity, and the release of cellular contents, which subsequently provoke an inflammatory response (Zhang H. et al., 2025). Although their mechanisms are distinct, both necrosis and cuproptosis may exhibit sensitivity to overlapping microenvironmental alterations. For instance, when the production of ROS overwhelms the cellular antioxidant defense capacity, it leads to oxidative stress. Oxidative stress is not only a key inducer of certain types of necrosis but may also indirectly interact with the cuproptosis process by impairing mitochondrial function (Ye et al., 2024).

Furthermore, intracellular glutathione (GSH) levels are a critical determinant influencing both cell death pathways. GSH exerts a dual role: it modulates susceptibility to cuproptosis and serves as a vital molecule in the cellular antioxidant system that defends against necrotic damage (Canli et al., 2016; Sun et al., 2025).

Cuproptosis and necrosis are fundamentally distinct in their mechanisms and morphological features. Cuproptosis is a novel, precisely regulated form of RCD driven by excess copper ions. Its central mechanism involves the specific binding of copper to lipoylated proteins within mitochondria, disrupting the tricarboxylic acid cycle and triggering a unique proteotoxic stress response. This entire process is underpinned by well-defined molecular targets and signaling pathways. In contrast, classical necrosis is generally considered a non-programmed, passive cell death process, primarily caused by severe physical, chemical, or pathological insults (e.g., extreme temperature, toxins, or ischemia) that directly induce irreversible loss of plasma membrane integrity. This is accompanied by organelle swelling and the release of cellular contents, provoking a strong inflammatory response, and lacks a specific, regulated molecular cascade. In summary, cuproptosis represents an active, metal ion-mediated signaling process, whereas necrosis is a passive cellular disintegration resulting from severe damage.

## 4 Potential therapeutic strategies targeting copper homeostasis imbalance in SCM

### 4.1 Copper overload and the inflammatory-oxidative stress axis

During sepsis, systemic inflammation can elevate serum copper and ceruloplasmin levels (Zhang W. et al., 2025). This copper overload engages in a vicious cycle with inflammation. On one hand, copper catalyzes the massive generation of ROS via the Fenton reaction, triggering oxidative stress. This process activates pro-inflammatory pathways such as NF-κB, promoting the release of cytokines including IL-1β and TNF-α. On the other hand, the inflammatory response itself further disrupts copper homeostasis. Mitochondria, serving as both a primary source and a key target of ROS, suffer impaired membrane potential and function during this process, exacerbating the cellular energy crisis (Jian et al., 2020; Petris and Mercer, 1999).

Copper chelators represent a therapeutic strategy that directly targets this upstream pathological nexus. They function by specifically binding and sequestering excess free copper ions, thereby cutting off the feed-forward cycle of oxidative stress and inflammatory amplification at its source. For instance, triethylenetetramine (TETA) has been shown in models of diabetic cardiomyopathy to restore myocardial antioxidant defenses and improve mitochondrial function through copper chelation, thereby exerting cardioprotective effects (Liu et al., 2018; Zhang et al., 2013; Zhang et al., 2020).

Similarly, TTM possesses not only chelating capacity but also anti-angiogenic and anti-inflammatory properties, potentially intervening concurrently against both copper overload and its downstream inflammatory consequences (Brewer, 2014; Medici and Sturniolo, 2008).

### 4.2 Copper transport and mitochondrial dysfunction

Copper enters cells and mitochondria via transporters such as CTR1, which is essential for maintaining the activity of enzymes including cytochrome c oxidase. However, dysregulated transport leading to abnormal copper accumulation within mitochondria directly inhibits the electron transport chain, disrupts the mitochondrial membrane potential, and induces the pathological opening of the mPTP. This cascade results in ATP depletion and activation of apoptotic signaling. Copper chaperone proteins, such as ATOX1 and CCS, play a critical regulatory role in this trafficking process (Durand et al., 2017; Garza et al., 2023; Oliveri, 2020; Pivovarova and Andrews, 2010; Tan et al., 2019; Tian et al., 2023).

Small-molecule inhibitors, exemplified by DC-AC50, offer a targeted strategy by specifically interfering with the function of copper chaperones ATOX1 and CCS. This blockade prevents the proper delivery of copper to client proteins like SOD1. Such disruption not only compromises cellular redox balance but also provides a novel rationale for precisely modulating intracellular copper distribution in cardiomyocytes (Wang et al., 2015).

Copper ionophores/complexes represent an alternative approach that functions by “reprogramming” copper distribution. The disulfiram (DSF)-copper complex, for instance, promotes ROS generation and inhibits NF-κB (Allensworth et al., 2015). Cu^II^(atsm) can act as a copper-delivering prodrug, releasing copper under specific pathological conditions (Huuskonen et al., 2017). More advanced targeted ionophores, such as Gal-Cu, enable organ-directed copper delivery, significantly minimizing off-target effects and heralding a new direction for precision modulation (Su et al., 2018).

### 4.3 Modulation of cell death

Recent studies have elucidated distinct forms of cell death induced by copper. Cuproptosis is characterized by the direct binding of excess copper to lipoylated enzymes in the TCA cycle, leading to their oligomerization and functional loss, which triggers irreversible mitochondrial metabolic collapse.

In parallel, ferroptosis is an iron-dependent form of cell death driven by the accumulation of lipid peroxides and the inactivation of GPX4. Copper overload contributes to both pathways: it promotes mitochondrial dysfunction and ROS bursts, while copper ions can also directly bind to and induce the degradation of GPX4, thereby orchestrating a synergistic promotion of ferroptosis.

Inducers/Modulators represent strategies designed to harness these death pathways for eliminating diseased cells. The copper ionophore Elesclomol efficiently shuttles copper into mitochondria, inducing cuproptosis in cancer cells (Modica-Napolitano et al., 2019). Conversely, the natural flavonoid Fisetin suppresses ferroptosis by upregulating GPX4 (Li D. et al., 2022). Its demonstrated protective effects in models of cardiac hypertrophy suggest its potential as a therapeutic agent for SCM.

Nanopreparations offer advanced platforms for spatially controlled intervention. Nanodrugs such as NP@ESCu are engineered to efficiently induce cuproptosis at target sites (Guo et al., 2023). The nanoliposomal composite Lipo-Ele@CuO_2_ co-delivers copper ions and the chelator elesclomol, inducing mitochondrial cuproptosis while synergistically enhancing radiotherapy efficacy (Jiang et al., 2025).

Meanwhile, PCEF@Fe employs a cooperative strategy: it chelates copper to weaken cellular defenses while simultaneously releasing iron to initiate ferroptosis, illustrating a novel approach for the coordinated targeting of multiple cell death pathways (Hua et al., 2025).

### 4.4 Multi-target therapeutic strategies for copper dyshomeostasis

Copper dyshomeostasis further disrupts other critical cellular homeostatic processes, including autophagy, ERS. Copper can modulate autophagic flux by activating the AMPK/mTOR pathway or by inducing oxidative stress; it can also trigger ERS by disrupting redox. The dysregulation of these processes collectively exacerbates cardiomyocyte injury and functional impairment.

Natural Products: Many natural compounds exhibit multi-target regulatory effects. Curcumin not only chelates copper but also possesses potent antioxidant and anti-inflammatory properties, potentially alleviating oxidative stress, ERS, and inflammation simultaneously (Baum and Ng, 2004; Zhang and Kitts, 2021; Zhang et al., 2016). Triptolide can disrupt copper homeostasis to induce cuproptosis in cancer cells, suggesting its potential to modulate cell fate pathways (Xiao et al., 2024).

Nanopreparations: Nanotechnology provides an ideal platform for integrating multi-mechanistic therapies. For instance, a nanocomposite that combines copper sulfide (CuS)-based photothermal therapy with anti-atherosclerotic chemotherapy enables precise drug delivery in response to the inflammatory microenvironment (Liu S. et al., 2022). This represents an advanced direction for synergistic therapy (Table 1).

**TABLE 1:** Core pathological axes and corresponding therapeutic interventions for copper dyshomeostasis in SCM.

Core targeted mechanism	Key processes and targets	Representative therapeutic strategies	Mechanism of action and therapeutic goal	References
Systemic copper overload and the inflammatory-oxidative stress axis	Free Cu^2+^, Fenton reaction, NF-κB pathway, inflammatory cytokines	Copper Chelators (e.g., TETA, TTM)	Directly sequester excess copper to disrupt the oxidative stress-inflammation cycle and mitigate systemic injury	Brewer (2014), Liu et al. (2018), Medici and Sturniolo (2008), Zhang et al. (2013), Zhang et al. (2020)
Copper transport and mitochondrial dysfunction	Copper transporters (e.g., CTR1), copper chaperones (e.g., ATOX1, CCS), mitochondrial copper homeostasis, mPTP	1. Small-Molecule Inhibitors (e.g., DC-AC50) 2. (Targeted) Copper Ionophores/Complexes (e.g., DSF, Cu(atsm), Gal-Cu)	1. Precisely interfere with intracellular copper transport and distribution 2. Reprogram subcellular copper distribution or achieve targeted delivery to restore/perturb mitochondrial function	Allensworth et al. (2015), Huuskonen et al. (2017), Su et al. (2018), Wang et al. (2015)
Modulation of cell death	TCA cycle, Fe-S clusters, GPX4, lipid ROS	1. Cell Death Pathway Inducers (e.g., Elesclomol) 2. Cell Death Pathway Inhibitors (e.g., Fisetin) 3. Multifunctional Nano-Platforms (e.g., NP@ESCu, Lipo-Ele@CuO_2_, PCEF@Fe)	1. Actively induce mitochondrial metabolic collapse (cuproptosis) 2. Enhance antioxidant defenses to suppress ferroptosis 3. Achieve precise delivery for coordinated regulation of multiple cell death pathways	Guo et al. (2023), Hua et al. (2025), Jiang et al. (2025), Li D. et al. (2022), Zheng et al. (2022)
Multi-target therapeutic strategies for copper dyshomeostasis	Autophagic flux, endoplasmic reticulum stress, calcium homeostasis	1. Multi-Target Natural Products (e.g., Curcumin, Triptolide) 2. Integrated Nanomedicines (e.g., CuS-based composites)	1. Indirectly stabilize the cellular microenvironment via antioxidant, anti-inflammatory, and stress-pathway modulatory effects 2. Enable multi-modal synergistic therapy integrating physical, chemical, and ionic regulation	Baum and Ng (2004), Liu S. et al. (2022), Xiao et al. (2024), Zhang and Kitts (2021), Zhang et al. (2016)

Current therapeutic strategies for myocardial injury based on copper primarily focus on two aspects: first, modulating copper homeostasis to prevent cuproptosis, and second, harnessing the biological properties of copper for therapeutic benefits. Copper chelator therapy involves the use of agents such as TTM to reduce excessive levels of free copper *in vivo*, thereby blocking copper-mediated pathological processes.

In a doxorubicin-induced cardiotoxicity model, treatment with TTM almost completely prevented the elevation of myocardial injury markers and inflammatory cytokines, demonstrating potent cardioprotective effects (Hou et al., 2005). Nanotherapeutic approaches utilize copper-based nanomaterials to mimic the activity of endogenous antioxidant enzymes, actively scavenging excess ROS to mitigate oxidative stress damage.

For example, a copper/manganese bimetallic nanozyme derived from a metal-organic framework exhibited superior SOD and catalase (CAT)-like activities in animal models of MI and I/R injury, effectively clearing ROS, reducing inflammation, and promoting the recovery of cardiac function (Xiang et al., 2023).

## 5 Conclusion and future perspectives

In recent years, the discovery of cuproptosis has significantly intensified research on copper-related cell death in cancer, cardiovascular, and immune diseases. Cu plays a dual role within organisms and cells: while it serves as an essential cofactor for numerous enzymes and participates in regulating vital biological processes, either an excess or a deficiency of Cu can trigger cell death and contribute to the pathogenesis of various diseases.

Cu influences cellular and systemic pathophysiology through multiple mechanisms, including oxidative stress, mitochondrial damage, autophagy, lipid metabolism, and inflammatory responses. Its effects vary across different cell types, reflecting context-specific roles. For instance, the interplay between oxidative stress and inflammation—often observed in Cu-induced cell death—has been implicated in the progression of CVD such as atherosclerosis, stroke, ischemia-reperfusion injury, heart failure, and hypertension.

However, the current understanding of the mechanisms of copper remains partially constrained by the lack of systematic findings and clinical validation in certain disease contexts. Moreover, copper homeostasis and its associated signaling pathways in SCM remain underexplored, with insufficient mechanistic studies to fully elucidate the relationship between copper dysregulation and disease. Further investigation is essential to clarify these aspects.

Notably, emerging evidence has established a compelling link between cuproptosis and SCM. Preliminary studies suggest that cuproptosis contributes to SCM progression, and Mettl1 as a novel suppressor of cuproptosis that confers protection against sepsis-induced cardiotoxicity by restraining FDX1-mediated copper-dependent cell death (Wei et al., 2026). Currently, most therapeutic approaches are primarily derived from cancer or Wilson’s disease research, leaving their application in SCM or other cardiovascular conditions largely unexplored.

Therefore, establishing reliable SCM models and subsequently determining precise therapeutic dosages and regimens is critical. A major focus for future clinical translation will be to enhance the design and application of targeted therapies while minimizing the toxic side effects associated with copper-targeting agents. Critical gaps remain, including the undefined optimal concentration of copper across various organs and cell types, likely due to variations in experimental models and conditions Establishing these reference values will provide a critical foundation for treating copper homeostasis-related disorders.

Furthermore, the mechanisms underlying existing copper-modulating drugs are still not fully understood. It is therefore worthwhile to explore innovative strategies—such as integrating high-throughput functional screening and artificial intelligence—to accelerate drug discovery and improve therapeutic precision. Research on copper homeostasis in SCM s advancing, yet several key questions remain unanswered.

One such question pertains to the necessity of a definitive marker to diagnose and confirm copper-related death resulting from dysregulation of copper homeostasis, and whether these markers may vary across different cells and organs. Furthermore, while numerous studies have established a connection between copper homeostasis and neurodegenerative diseases, the potential therapeutic utility of inhibiting copper-related death in these conditions warrants further investigation.

Additionally, exploring the phenotype of cell death triggered by copper homeostasis imbalance, elucidating the mechanism by which aggregation of fatty acylated proteins initiates a cascade of cell death, understanding the role of copper in mitochondria, and investigating other potential roles of copper in endoplasmic reticulum stress necessitate further experimental research.

Ultimately, addressing these inquiries will enhance our comprehension of the interplay between copper homeostasis and SCM, thereby informing future therapeutic interventions and advancing research on genetic and hereditary copper-related diseases.
