# Correction: Mechanisms and therapeutic strategies of copper homeostasis in the pathogenesis of sepsis-induced cardiomyopathy

**DOI:** 10.3389/fcell.2026.1823928

**Published:** 2026-03-16

**Authors:** Zihao Xie, He Wang, Bohua You, Mengmeng Li, Duo Li, Huaying Wu

**Affiliations:** 1 Hunan Normal University Health Science Center, Changsha, Hunan, China; 2 Key Laboratory of Study and Discovery of Small Targeted Molecules of Hunan Province, Hunan Normal University Health Science Center, Changsha, Hunan, China; 3 Department of General Surgery, People’s Hospital of Honghu City, Honghu, Hubei, China

**Keywords:** cell death, copper homeostasis, cuproptosis, sepsis, sepsis-induced cardiomyopathy

The **funder** [National Natural Science Foundation of China], [No. 82405355] to [Huaying Wu] was erroneously omitted.

The original article has been updated.

